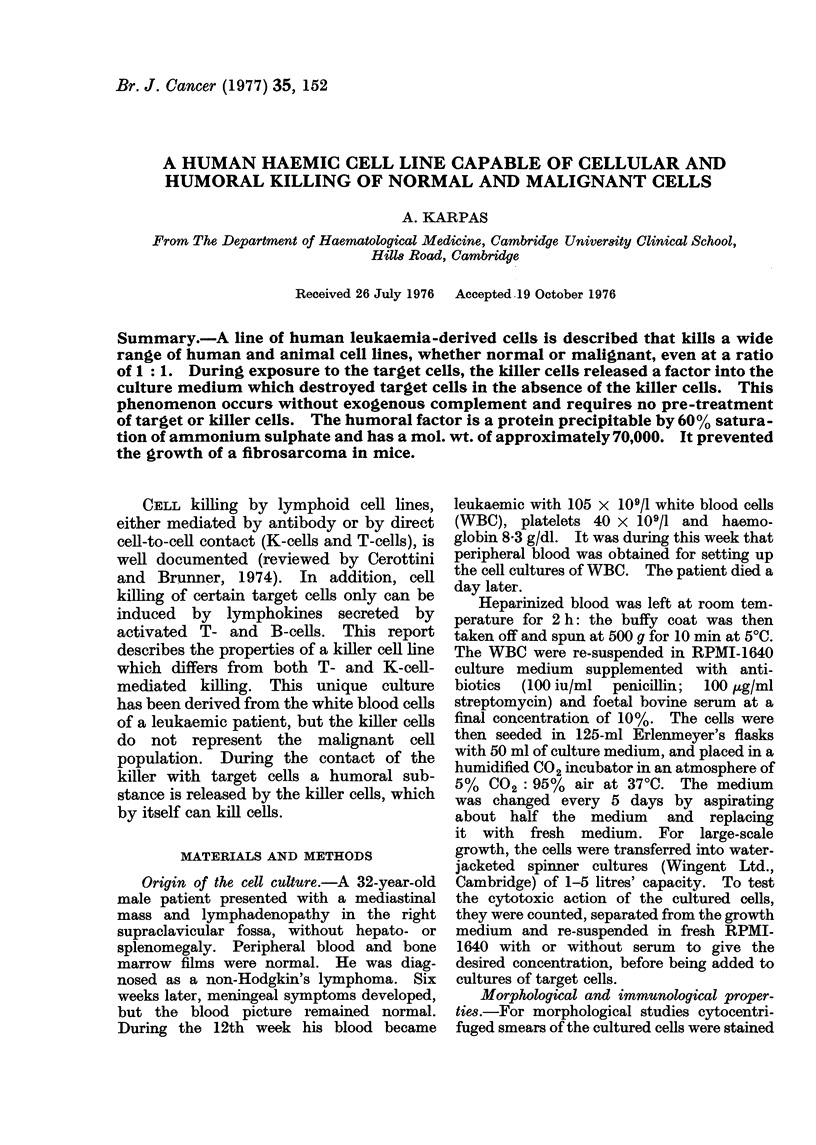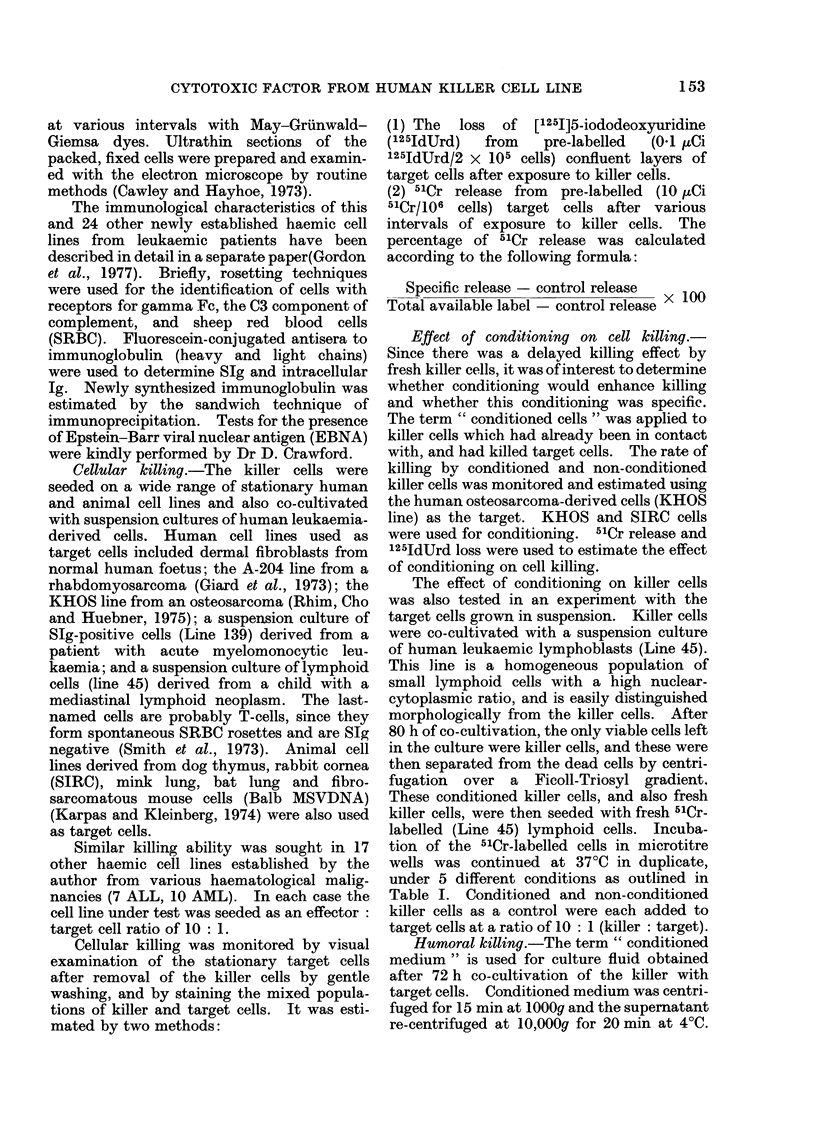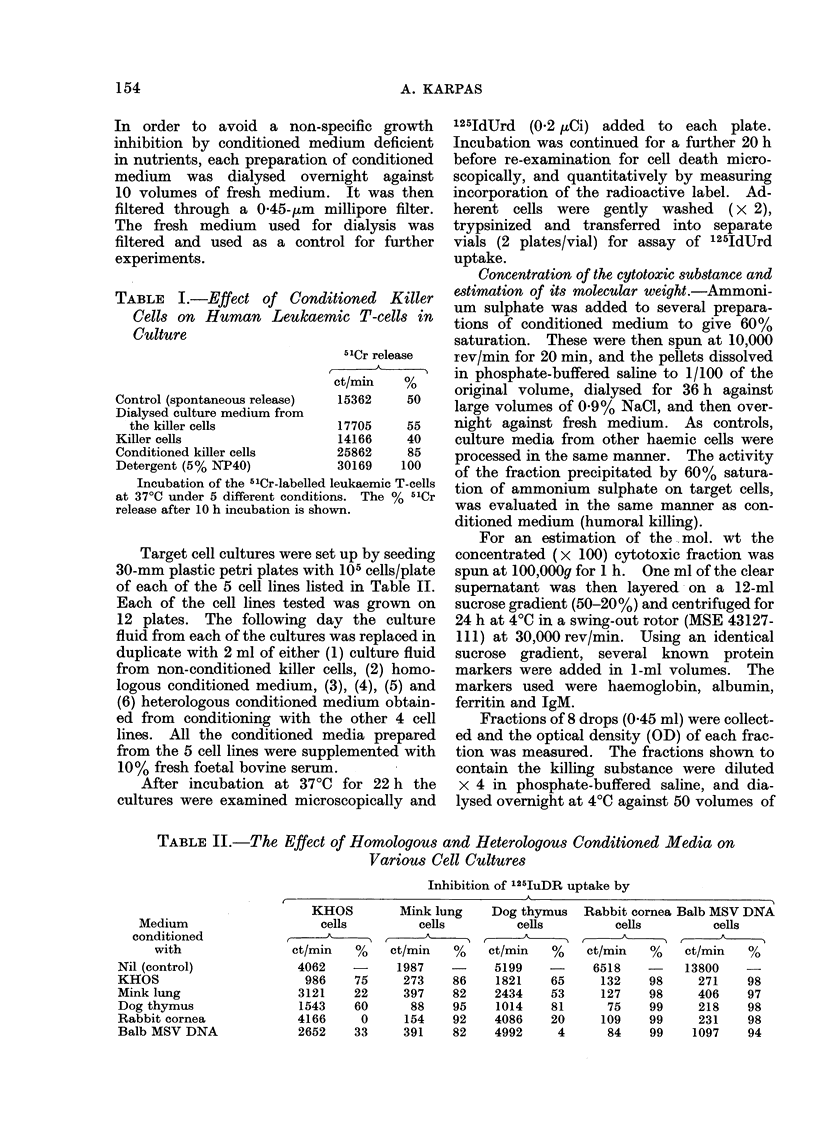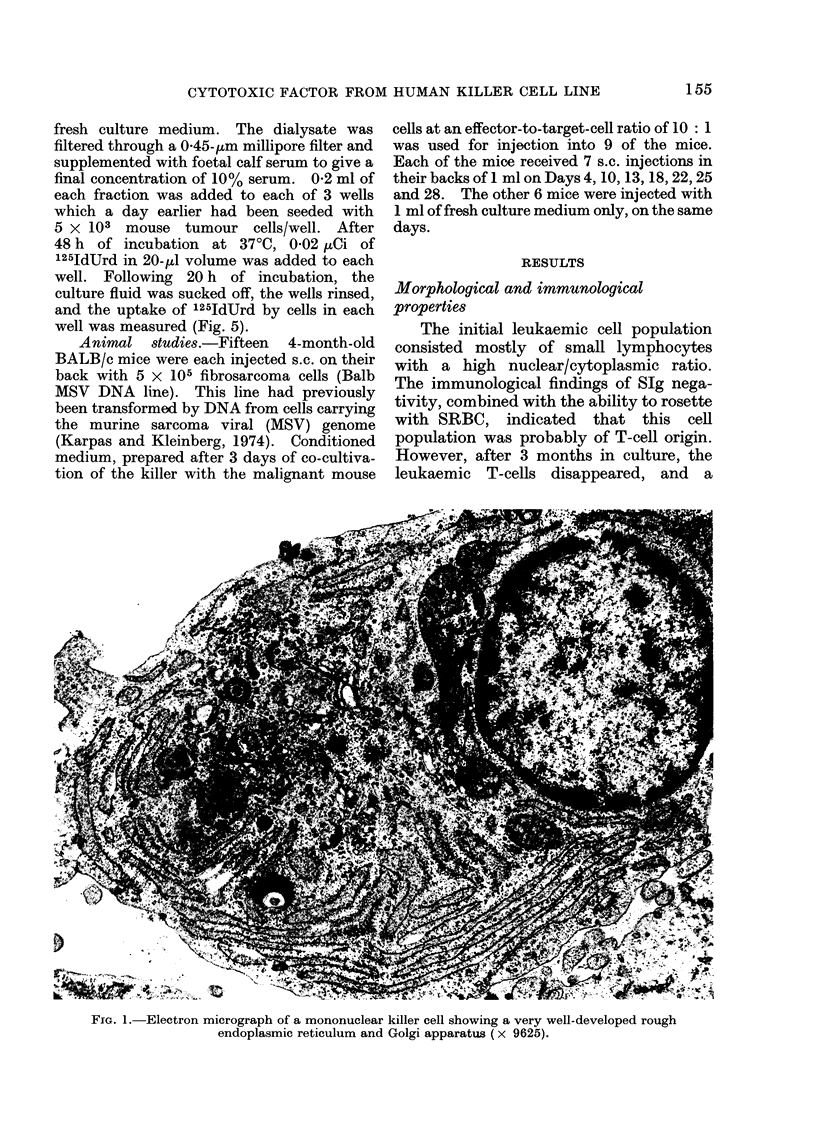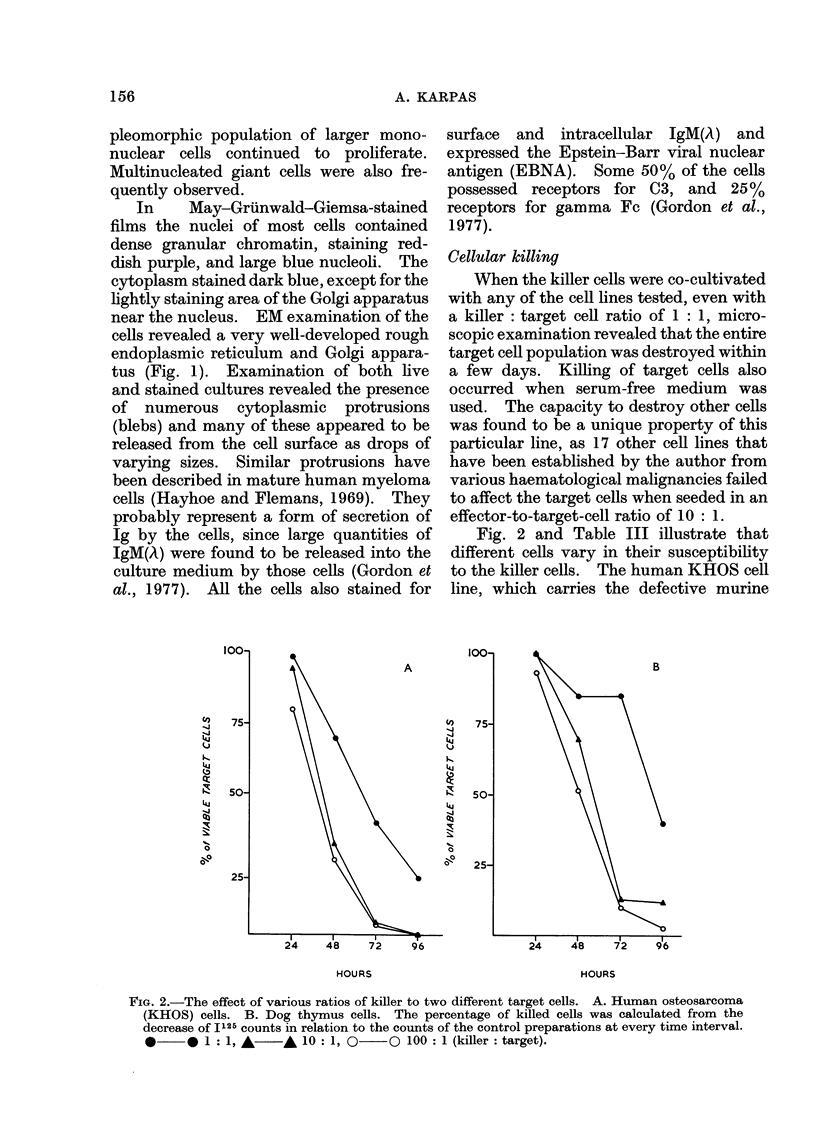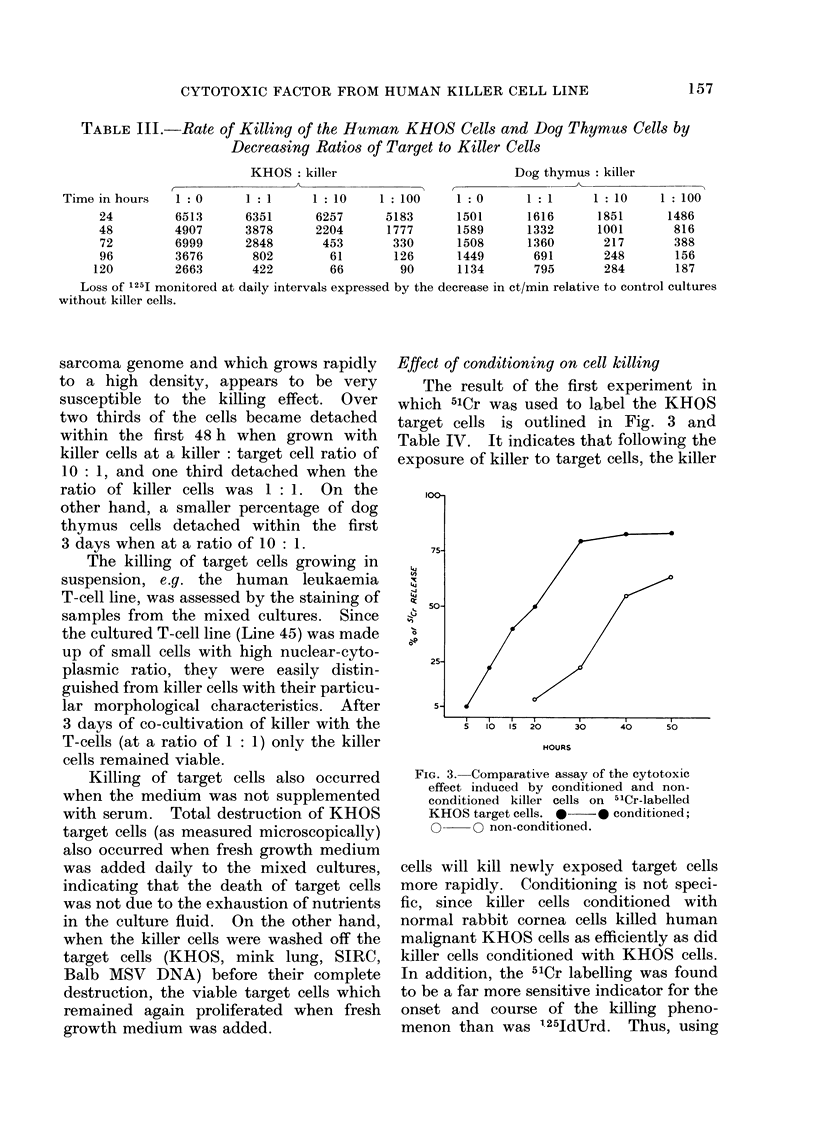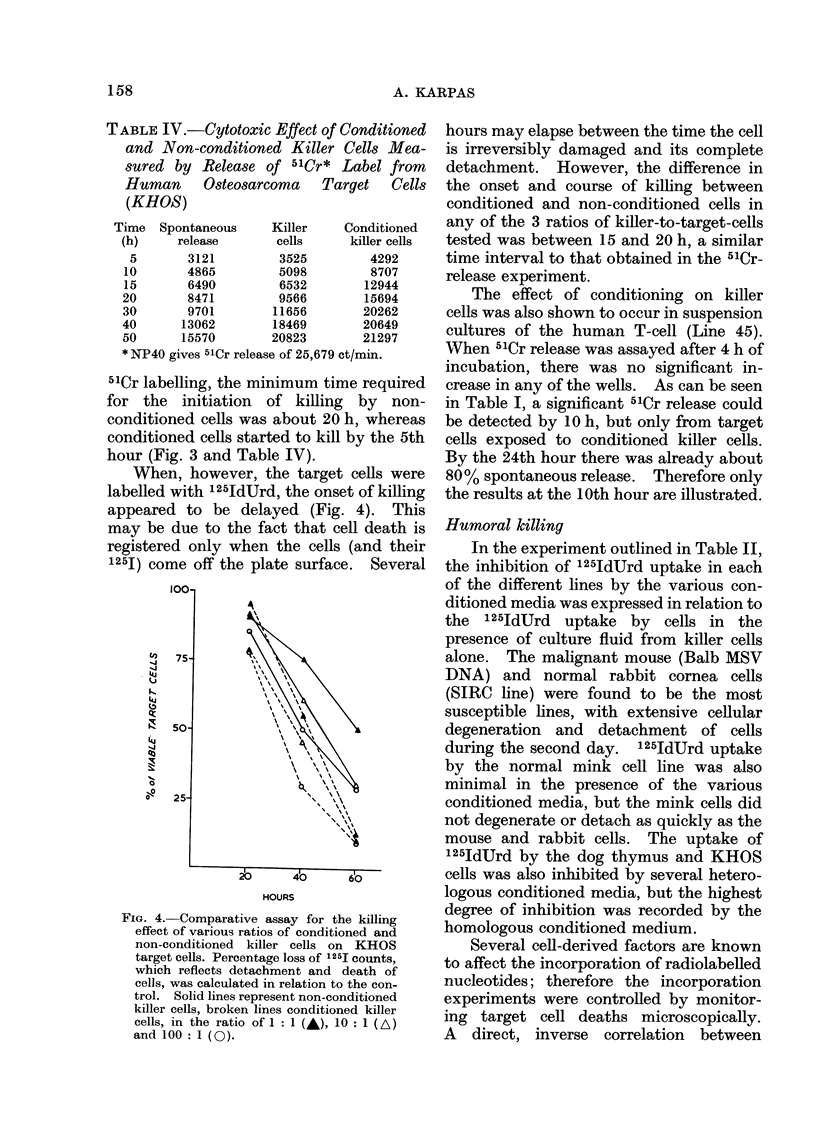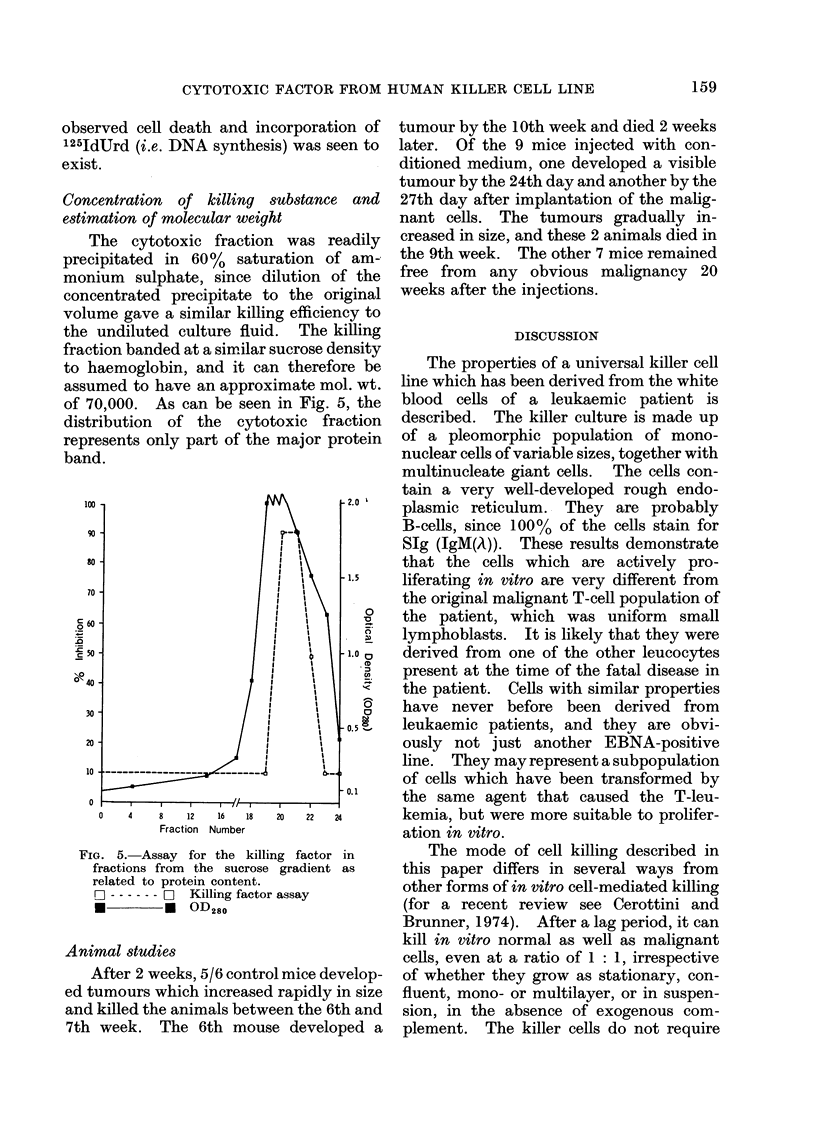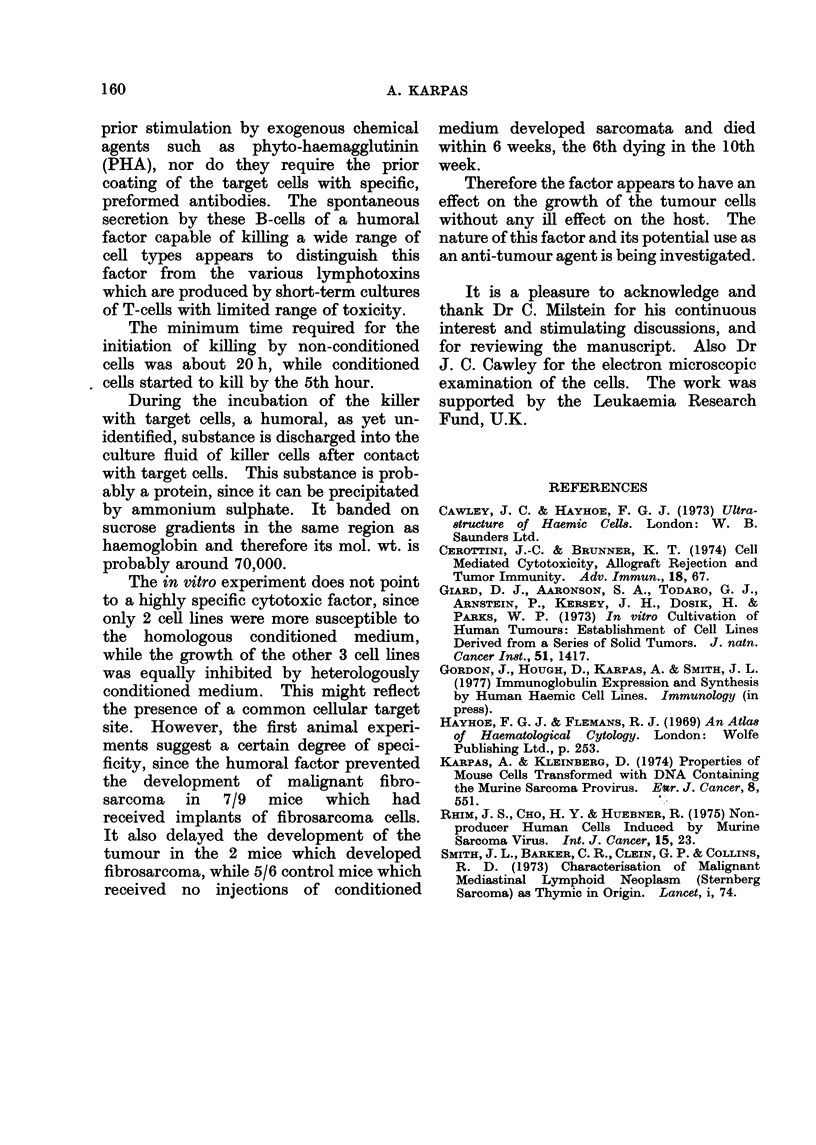# A human haemic cell line capable of cellular and humoral killing of normal and malignant cells.

**DOI:** 10.1038/bjc.1977.22

**Published:** 1977-02

**Authors:** A. Karpas

## Abstract

**Images:**


*Br. J. Cancer* (1977) **35**, 152

### A HUMAN HAEMIC CELL LINE CAPABLE OF CELLULAR AND HUMORAL KILLING OF NORMAL AND MALIGNANT CELLS

A. KARPAS

*From The Department of Haematological Medicine, Cambridge University Clinical School, Hills Road, Cambridge*

Received 26 July 1976 Accepted 19 October 1976

**Summary.—A line of human leukaemia-derived cells is described that kills a wide range of human and animal cell lines, whether normal or malignant, even at a ratio of 1 : 1. During exposure to the target cells, the killer cells released a factor into the culture medium which destroyed target cells in the absence of the killer cells. This phenomenon occurs without exogenous complement and requires no pre-treatment of target or killer cells. The humoral factor is a protein precipitable by 60% saturation of ammonium sulphate and has a mol. wt. of approximately 70,000. It prevented the growth of a fibrosarcoma in mice.**

CELL killing by lymphoid cell lines, either mediated by antibody or by direct cell-to-cell contact (K-cells and T-cells), is well documented (reviewed by Cerottini and Brunner, 1974). In addition, cell killing of certain target cells only can be induced by lymphokines secreted by activated T- and B-cells. This report describes the properties of a killer cell line which differs from both T- and K-cell-mediated killing. This unique culture has been derived from the white blood cells of a leukaemic patient, but the killer cells do not represent the malignant cell population. During the contact of the killer with target cells a humoral substance is released by the killer cells, which by itself can kill cells.

### MATERIALS AND METHODS

*Origin of the cell culture.*—A 32-year-old male patient presented with a mediastinal mass and lymphadenopathy in the right supraclavicular fossa, without hepato- or splenomegaly. Peripheral blood and bone marrow films were normal. He was diagnosed as a non-Hodgkin's lymphoma. Six weeks later, meningeal symptoms developed, but the blood picture remained normal. During the 12th week his blood became leukaemic with 105 × $10^9$/l white blood cells (WBC), platelets 40 × $10^9$/l and haemoglobin 8·3 g/dl. It was during this week that peripheral blood was obtained for setting up the cell cultures of WBC. The patient died a day later.

Heparinized blood was left at room temperature for 2 h: the buffy coat was then taken off and spun at 500 *g* for 10 min at 5°C. The WBC were re-suspended in RPMI-1640 culture medium supplemented with antibiotics (100 iu/ml penicillin; 100 μg/ml streptomycin) and foetal bovine serum at a final concentration of 10%. The cells were then seeded in 125-ml Erlenmeyer's flasks with 50 ml of culture medium, and placed in a humidified $CO_2$ incubator in an atmosphere of 5% $CO_2$ : 95% air at 37°C. The medium was changed every 5 days by aspirating about half the medium and replacing it with fresh medium. For large-scale growth, the cells were transferred into water-jacketed spinner cultures (Wingent Ltd., Cambridge) of 1–5 litres' capacity. To test the cytotoxic action of the cultured cells, they were counted, separated from the growth medium and re-suspended in fresh RPMI-1640 with or without serum to give the desired concentration, before being added to cultures of target cells.

*Morphological and immunological properties.*—For morphological studies cytocentrifuged smears of the cultured cells were stained at various intervals with May–Grünwald–Giemsa dyes. Ultrathin sections of the packed, fixed cells were prepared and examined with the electron microscope by routine methods (Cawley and Hayhoe, 1973).

The immunological characteristics of this and 24 other newly established haemic cell lines from leukaemic patients have been described in detail in a separate paper (Gordon *et al.*, 1977). Briefly, rosetting techniques were used for the identification of cells with receptors for gamma Fc, the C3 component of complement, and sheep red blood cells (SRBC). Fluorescein-conjugated antisera to immunoglobulin (heavy and light chains) were used to determine SIg and intracellular Ig. Newly synthesized immunoglobulin was estimated by the sandwich technique of immunoprecipitation. Tests for the presence of Epstein–Barr viral nuclear antigen (EBNA) were kindly performed by Dr D. Crawford.

*Cellular killing*.—The killer cells were seeded on a wide range of stationary human and animal cell lines and also co-cultivated with suspension cultures of human leukaemia-derived cells. Human cell lines used as target cells included dermal fibroblasts from normal human foetus; the A-204 line from a rhabdomyosarcoma (Giard *et al.*, 1973); the KHOS line from an osteosarcoma (Rhim, Cho and Huebner, 1975); a suspension culture of SIg-positive cells (Line 139) derived from a patient with acute myelomonocytic leukaemia; and a suspension culture of lymphoid cells (line 45) derived from a child with a mediastinal lymphoid neoplasm. The last-named cells are probably T-cells, since they form spontaneous SRBC rosettes and are SIg negative (Smith *et al.*, 1973). Animal cell lines derived from dog thymus, rabbit cornea (SIRC), mink lung, bat lung and fibrosarcomatous mouse cells (Balb MSVDNA) (Karpas and Kleinberg, 1974) were also used as target cells.

Similar killing ability was sought in 17 other haemic cell lines established by the author from various haematological malignancies (7 ALL, 10 AML). In each case the cell line under test was seeded as an effector : target cell ratio of 10 : 1.

Cellular killing was monitored by visual examination of the stationary target cells after removal of the killer cells by gentle washing, and by staining the mixed populations of killer and target cells. It was estimated by two methods:

(1) The loss of [$^{125}$I]5-iododeoxyuridine ($^{125}$IdUrd) from pre-labelled (0·1 $\mu$Ci $^{125}$IdUrd/2 $\times$ $10^5$ cells) confluent layers of target cells after exposure to killer cells.
(2) $^{51}$Cr release from pre-labelled (10 $\mu$Ci $^{51}$Cr/$10^6$ cells) target cells after various intervals of exposure to killer cells. The percentage of $^{51}$Cr release was calculated according to the following formula:

$$\frac{\text{Specific release} - \text{control release}}{\text{Total available label} - \text{control release}} \times 100$$

*Effect of conditioning on cell killing*.—Since there was a delayed killing effect by fresh killer cells, it was of interest to determine whether conditioning would enhance killing and whether this conditioning was specific. The term " conditioned cells " was applied to killer cells which had already been in contact with, and had killed target cells. The rate of killing by conditioned and non-conditioned killer cells was monitored and estimated using the human osteosarcoma-derived cells (KHOS line) as the target. KHOS and SIRC cells were used for conditioning. $^{51}$Cr release and $^{125}$IdUrd loss were used to estimate the effect of conditioning on cell killing.

The effect of conditioning on killer cells was also tested in an experiment with the target cells grown in suspension. Killer cells were co-cultivated with a suspension culture of human leukaemic lymphoblasts (Line 45). This line is a homogeneous population of small lymphoid cells with a high nuclear-cytoplasmic ratio, and is easily distinguished morphologically from the killer cells. After 80 h of co-cultivation, the only viable cells left in the culture were killer cells, and these were then separated from the dead cells by centrifugation over a Ficoll-Triosyl gradient. These conditioned killer cells, and also fresh killer cells, were then seeded with fresh $^{51}$Cr-labelled (Line 45) lymphoid cells. Incubation of the $^{51}$Cr-labelled cells in microtitre wells was continued at 37°C in duplicate, under 5 different conditions as outlined in Table I. Conditioned and non-conditioned killer cells as a control were each added to target cells at a ratio of 10 : 1 (killer : target).

*Humoral killing*.—The term " conditioned medium " is used for culture fluid obtained after 72 h co-cultivation of the killer with target cells. Conditioned medium was centrifuged for 15 min at 1000$g$ and the supernatant re-centrifuged at 10,000$g$ for 20 min at 4°C.

In order to avoid a non-specific growth inhibition by conditioned medium deficient in nutrients, each preparation of conditioned medium was dialysed overnight against 10 volumes of fresh medium. It was then filtered through a 0·45-$\mu$m millipore filter. The fresh medium used for dialysis was filtered and used as a control for further experiments.

TABLE I.—*Effect of Conditioned Killer Cells on Human Leukaemic T-cells in Culture*

| | $^{51}$Cr release | |
|---|---|---|
| | ct/min | % |
| Control (spontaneous release) | 15362 | 50 |
| Dialysed culture medium from the killer cells | 17705 | 55 |
| Killer cells | 14166 | 40 |
| Conditioned killer cells | 25862 | 85 |
| Detergent (5% NP40) | 30169 | 100 |

Incubation of the $^{51}$Cr-labelled leukaemic T-cells at 37°C under 5 different conditions. The % $^{51}$Cr release after 10 h incubation is shown.

Target cell cultures were set up by seeding 30-mm plastic petri plates with $10^5$ cells/plate of each of the 5 cell lines listed in Table II. Each of the cell lines tested was grown on 12 plates. The following day the culture fluid from each of the cultures was replaced in duplicate with 2 ml of either (1) culture fluid from non-conditioned killer cells, (2) homologous conditioned medium, (3), (4), (5) and (6) heterologous conditioned medium obtained from conditioning with the other 4 cell lines. All the conditioned media prepared from the 5 cell lines were supplemented with 10% fresh foetal bovine serum.

After incubation at 37°C for 22 h the cultures were examined microscopically and $^{125}$IdUrd (0·2 $\mu$Ci) added to each plate. Incubation was continued for a further 20 h before re-examination for cell death microscopically, and quantitatively by measuring incorporation of the radioactive label. Adherent cells were gently washed (× 2), trypsinized and transferred into separate vials (2 plates/vial) for assay of $^{125}$IdUrd uptake.

*Concentration of the cytotoxic substance and estimation of its molecular weight.*—Ammonium sulphate was added to several preparations of conditioned medium to give 60% saturation. These were then spun at 10,000 rev/min for 20 min, and the pellets dissolved in phosphate-buffered saline to 1/100 of the original volume, dialysed for 36 h against large volumes of 0·9% NaCl, and then overnight against fresh medium. As controls, culture media from other haemic cells were processed in the same manner. The activity of the fraction precipitated by 60% saturation of ammonium sulphate on target cells, was evaluated in the same manner as conditioned medium (humoral killing).

For an estimation of the mol. wt the concentrated (× 100) cytotoxic fraction was spun at 100,000$g$ for 1 h. One ml of the clear supernatant was then layered on a 12-ml sucrose gradient (50–20%) and centrifuged for 24 h at 4°C in a swing-out rotor (MSE 43127-111) at 30,000 rev/min. Using an identical sucrose gradient, several known protein markers were added in 1-ml volumes. The markers used were haemoglobin, albumin, ferritin and IgM.

Fractions of 8 drops (0·45 ml) were collected and the optical density (OD) of each fraction was measured. The fractions shown to contain the killing substance were diluted × 4 in phosphate-buffered saline, and dialysed overnight at 4°C against 50 volumes of

TABLE II.—*The Effect of Homologous and Heterologous Conditioned Media on Various Cell Cultures*

| Medium conditioned with | Inhibition of $^{125}$IuDR uptake by: KHOS cells | | Mink lung cells | | Dog thymus cells | | Rabbit cornea cells | | Balb MSV DNA cells | |
|---|---|---|---|---|---|---|---|---|---|---|
| | ct/min | % | ct/min | % | ct/min | % | ct/min | % | ct/min | % |
| Nil (control) | 4062 | — | 1987 | — | 5199 | — | 6518 | — | 13800 | — |
| KHOS | 986 | 75 | 273 | 86 | 1821 | 65 | 132 | 98 | 271 | 98 |
| Mink lung | 3121 | 22 | 397 | 82 | 2434 | 53 | 127 | 98 | 406 | 97 |
| Dog thymus | 1543 | 60 | 88 | 95 | 1014 | 81 | 75 | 99 | 218 | 98 |
| Rabbit cornea | 4166 | 0 | 154 | 92 | 4086 | 20 | 109 | 99 | 231 | 98 |
| Balb MSV DNA | 2652 | 33 | 391 | 82 | 4992 | 4 | 84 | 99 | 1097 | 94 |

fresh culture medium. The dialysate was filtered through a 0·45-$\mu$m millipore filter and supplemented with foetal calf serum to give a final concentration of 10% serum. 0·2 ml of each fraction was added to each of 3 wells which a day earlier had been seeded with $5 \times 10^3$ mouse tumour cells/well. After 48 h of incubation at 37°C, 0·02 $\mu$Ci of $^{125}$IdUrd in 20-$\mu$l volume was added to each well. Following 20 h of incubation, the culture fluid was sucked off, the wells rinsed, and the uptake of $^{125}$IdUrd by cells in each well was measured (Fig. 5).

*Animal studies.*—Fifteen 4-month-old BALB/c mice were each injected s.c. on their back with $5 \times 10^5$ fibrosarcoma cells (Balb MSV DNA line). This line had previously been transformed by DNA from cells carrying the murine sarcoma viral (MSV) genome (Karpas and Kleinberg, 1974). Conditioned medium, prepared after 3 days of co-cultivation of the killer with the malignant mouse cells at an effector-to-target-cell ratio of 10 : 1 was used for injection into 9 of the mice. Each of the mice received 7 s.c. injections in their backs of 1 ml on Days 4, 10, 13, 18, 22, 25 and 28. The other 6 mice were injected with 1 ml of fresh culture medium only, on the same days.

### RESULTS

### *Morphological and immunological properties*

The initial leukaemic cell population consisted mostly of small lymphocytes with a high nuclear/cytoplasmic ratio. The immunological findings of SIg negativity, combined with the ability to rosette with SRBC, indicated that this cell population was probably of T-cell origin. However, after 3 months in culture, the leukaemic T-cells disappeared, and a

FIG. 1.—Electron micrograph of a mononuclear killer cell showing a very well-developed rough endoplasmic reticulum and Golgi apparatus (× 9625).

pleomorphic population of larger mononuclear cells continued to proliferate. Multinucleated giant cells were also frequently observed.

In May–Grünwald–Giemsa-stained films the nuclei of most cells contained dense granular chromatin, staining reddish purple, and large blue nucleoli. The cytoplasm stained dark blue, except for the lightly staining area of the Golgi apparatus near the nucleus. EM examination of the cells revealed a very well-developed rough endoplasmic reticulum and Golgi apparatus (Fig. 1). Examination of both live and stained cultures revealed the presence of numerous cytoplasmic protrusions (blebs) and many of these appeared to be released from the cell surface as drops of varying sizes. Similar protrusions have been described in mature human myeloma cells (Hayhoe and Flemans, 1969). They probably represent a form of secretion of Ig by the cells, since large quantities of IgM(λ) were found to be released into the culture medium by those cells (Gordon *et al.*, 1977). All the cells also stained for surface and intracellular IgM(λ) and expressed the Epstein–Barr viral nuclear antigen (EBNA). Some 50% of the cells possessed receptors for C3, and 25% receptors for gamma Fc (Gordon *et al.*, 1977).

### *Cellular killing*

When the killer cells were co-cultivated with any of the cell lines tested, even with a killer : target cell ratio of 1 : 1, microscopic examination revealed that the entire target cell population was destroyed within a few days. Killing of target cells also occurred when serum-free medium was used. The capacity to destroy other cells was found to be a unique property of this particular line, as 17 other cell lines that have been established by the author from various haematological malignancies failed to affect the target cells when seeded in an effector-to-target-cell ratio of 10 : 1.

Fig. 2 and Table III illustrate that different cells vary in their susceptibility to the killer cells. The human KHOS cell line, which carries the defective murine

FIG. 2.—The effect of various ratios of killer to two different target cells. A. Human osteosarcoma (KHOS) cells. B. Dog thymus cells. The percentage of killed cells was calculated from the decrease of $I^{125}$ counts in relation to the counts of the control preparations at every time interval. ●——● 1 : 1, ▲——▲ 10 : 1, ○——○ 100 : 1 (killer : target).

TABLE III.—*Rate of Killing of the Human KHOS Cells and Dog Thymus Cells by Decreasing Ratios of Target to Killer Cells*

| | KHOS : killer | | | | Dog thymus : killer | | | |
|---|---|---|---|---|---|---|---|---|
| Time in hours | 1 : 0 | 1 : 1 | 1 : 10 | 1 : 100 | 1 : 0 | 1 : 1 | 1 : 10 | 1 : 100 |
| 24 | 6513 | 6351 | 6257 | 5183 | 1501 | 1616 | 1851 | 1486 |
| 48 | 4907 | 3878 | 2204 | 1777 | 1589 | 1332 | 1001 | 816 |
| 72 | 6999 | 2848 | 453 | 330 | 1508 | 1360 | 217 | 388 |
| 96 | 3676 | 802 | 61 | 126 | 1449 | 691 | 248 | 156 |
| 120 | 2663 | 422 | 66 | 90 | 1134 | 795 | 284 | 187 |

Loss of $^{125}I$ monitored at daily intervals expressed by the decrease in ct/min relative to control cultures without killer cells.

sarcoma genome and which grows rapidly to a high density, appears to be very susceptible to the killing effect. Over two thirds of the cells became detached within the first 48 h when grown with killer cells at a killer : target cell ratio of 10 : 1, and one third detached when the ratio of killer cells was 1 : 1. On the other hand, a smaller percentage of dog thymus cells detached within the first 3 days when at a ratio of 10 : 1.

The killing of target cells growing in suspension, *e.g.* the human leukaemia T-cell line, was assessed by the staining of samples from the mixed cultures. Since the cultured T-cell line (Line 45) was made up of small cells with high nuclear-cytoplasmic ratio, they were easily distinguished from killer cells with their particular morphological characteristics. After 3 days of co-cultivation of killer with the T-cells (at a ratio of 1 : 1) only the killer cells remained viable.

Killing of target cells also occurred when the medium was not supplemented with serum. Total destruction of KHOS target cells (as measured microscopically) also occurred when fresh growth medium was added daily to the mixed cultures, indicating that the death of target cells was not due to the exhaustion of nutrients in the culture fluid. On the other hand, when the killer cells were washed off the target cells (KHOS, mink lung, SIRC, Balb MSV DNA) before their complete destruction, the viable target cells which remained again proliferated when fresh growth medium was added.

*Effect of conditioning on cell killing*

The result of the first experiment in which $^{51}Cr$ was used to label the KHOS target cells is outlined in Fig. 3 and Table IV. It indicates that following the exposure of killer to target cells, the killer

FIG. 3.—Comparative assay of the cytotoxic effect induced by conditioned and non-conditioned killer cells on $^{51}Cr$-labelled KHOS target cells. ●——● conditioned; ○——○ non-conditioned.

cells will kill newly exposed target cells more rapidly. Conditioning is not specific, since killer cells conditioned with normal rabbit cornea cells killed human malignant KHOS cells as efficiently as did killer cells conditioned with KHOS cells. In addition, the $^{51}Cr$ labelling was found to be a far more sensitive indicator for the onset and course of the killing phenomenon than was $^{125}IdUrd$. Thus, using

TABLE IV.—*Cytotoxic Effect of Conditioned and Non-conditioned Killer Cells Measured by Release of $^{51}Cr$* Label from Human Osteosarcoma Target Cells (KHOS)*

| Time (h) | Spontaneous release | Killer cells | Conditioned killer cells |
|---|---|---|---|
| 5 | 3121 | 3525 | 4292 |
| 10 | 4865 | 5098 | 8707 |
| 15 | 6490 | 6532 | 12944 |
| 20 | 8471 | 9566 | 15694 |
| 30 | 9701 | 11656 | 20262 |
| 40 | 13062 | 18469 | 20649 |
| 50 | 15570 | 20823 | 21297 |

* NP40 gives $^{51}Cr$ release of 25,679 ct/min.

$^{51}Cr$ labelling, the minimum time required for the initiation of killing by non-conditioned cells was about 20 h, whereas conditioned cells started to kill by the 5th hour (Fig. 3 and Table IV).

When, however, the target cells were labelled with $^{125}IdUrd$, the onset of killing appeared to be delayed (Fig. 4). This may be due to the fact that cell death is registered only when the cells (and their $^{125}I$) come off the plate surface. Several

FIG. 4.—Comparative assay for the killing effect of various ratios of conditioned and non-conditioned killer cells on KHOS target cells. Percentage loss of $^{125}I$ counts, which reflects detachment and death of cells, was calculated in relation to the control. Solid lines represent non-conditioned killer cells, broken lines conditioned killer cells, in the ratio of 1 : 1 (▲), 10 : 1 (△) and 100 : 1 (○).

hours may elapse between the time the cell is irreversibly damaged and its complete detachment. However, the difference in the onset and course of killing between conditioned and non-conditioned cells in any of the 3 ratios of killer-to-target-cells tested was between 15 and 20 h, a similar time interval to that obtained in the $^{51}Cr$-release experiment.

The effect of conditioning on killer cells was also shown to occur in suspension cultures of the human T-cell (Line 45). When $^{51}Cr$ release was assayed after 4 h of incubation, there was no significant increase in any of the wells. As can be seen in Table I, a significant $^{51}Cr$ release could be detected by 10 h, but only from target cells exposed to conditioned killer cells. By the 24th hour there was already about 80% spontaneous release. Therefore only the results at the 10th hour are illustrated.

### *Humoral killing*

In the experiment outlined in Table II, the inhibition of $^{125}IdUrd$ uptake in each of the different lines by the various conditioned media was expressed in relation to the $^{125}IdUrd$ uptake by cells in the presence of culture fluid from killer cells alone. The malignant mouse (Balb MSV DNA) and normal rabbit cornea cells (SIRC line) were found to be the most susceptible lines, with extensive cellular degeneration and detachment of cells during the second day. $^{125}IdUrd$ uptake by the normal mink cell line was also minimal in the presence of the various conditioned media, but the mink cells did not degenerate or detach as quickly as the mouse and rabbit cells. The uptake of $^{125}IdUrd$ by the dog thymus and KHOS cells was also inhibited by several heterologous conditioned media, but the highest degree of inhibition was recorded by the homologous conditioned medium.

Several cell-derived factors are known to affect the incorporation of radiolabelled nucleotides; therefore the incorporation experiments were controlled by monitoring target cell deaths microscopically. A direct, inverse correlation between observed cell death and incorporation of [125]IdUrd (*i.e.* DNA synthesis) was seen to exist.

*Concentration of killing substance and estimation of molecular weight*

The cytotoxic fraction was readily precipitated in 60% saturation of ammonium sulphate, since dilution of the concentrated precipitate to the original volume gave a similar killing efficiency to the undiluted culture fluid. The killing fraction banded at a similar sucrose density to haemoglobin, and it can therefore be assumed to have an approximate mol. wt. of 70,000. As can be seen in Fig. 5, the distribution of the cytotoxic fraction represents only part of the major protein band.

FIG. 5.—Assay for the killing factor in fractions from the sucrose gradient as related to protein content.

□ - - - - - - □ Killing factor assay

■———■ $OD_{280}$

*Animal studies*

After 2 weeks, 5/6 control mice developed tumours which increased rapidly in size and killed the animals between the 6th and 7th week. The 6th mouse developed a tumour by the 10th week and died 2 weeks later. Of the 9 mice injected with conditioned medium, one developed a visible tumour by the 24th day and another by the 27th day after implantation of the malignant cells. The tumours gradually increased in size, and these 2 animals died in the 9th week. The other 7 mice remained free from any obvious malignancy 20 weeks after the injections.

### DISCUSSION

The properties of a universal killer cell line which has been derived from the white blood cells of a leukaemic patient is described. The killer culture is made up of a pleomorphic population of mononuclear cells of variable sizes, together with multinucleate giant cells. The cells contain a very well-developed rough endoplasmic reticulum. They are probably B-cells, since 100% of the cells stain for SIg (IgM(λ)). These results demonstrate that the cells which are actively proliferating *in vitro* are very different from the original malignant T-cell population of the patient, which was uniform small lymphoblasts. It is likely that they were derived from one of the other leucocytes present at the time of the fatal disease in the patient. Cells with similar properties have never before been derived from leukaemic patients, and they are obviously not just another EBNA-positive line. They may represent a subpopulation of cells which have been transformed by the same agent that caused the T-leukemia, but were more suitable to proliferation *in vitro*.

The mode of cell killing described in this paper differs in several ways from other forms of *in vitro* cell-mediated killing (for a recent review see Cerottini and Brunner, 1974). After a lag period, it can kill *in vitro* normal as well as malignant cells, even at a ratio of 1 : 1, irrespective of whether they grow as stationary, confluent, mono- or multilayer, or in suspension, in the absence of exogenous complement. The killer cells do not require prior stimulation by exogenous chemical agents such as phyto-haemagglutinin (PHA), nor do they require the prior coating of the target cells with specific, preformed antibodies. The spontaneous secretion by these B-cells of a humoral factor capable of killing a wide range of cell types appears to distinguish this factor from the various lymphotoxins which are produced by short-term cultures of T-cells with limited range of toxicity.

The minimum time required for the initiation of killing by non-conditioned cells was about 20 h, while conditioned cells started to kill by the 5th hour.

During the incubation of the killer with target cells, a humoral, as yet unidentified, substance is discharged into the culture fluid of killer cells after contact with target cells. This substance is probably a protein, since it can be precipitated by ammonium sulphate. It banded on sucrose gradients in the same region as haemoglobin and therefore its mol. wt. is probably around 70,000.

The *in vitro* experiment does not point to a highly specific cytotoxic factor, since only 2 cell lines were more susceptible to the homologous conditioned medium, while the growth of the other 3 cell lines was equally inhibited by heterologously conditioned medium. This might reflect the presence of a common cellular target site. However, the first animal experiments suggest a certain degree of specificity, since the humoral factor prevented the development of malignant fibrosarcoma in 7/9 mice which had received implants of fibrosarcoma cells. It also delayed the development of the tumour in the 2 mice which developed fibrosarcoma, while 5/6 control mice which received no injections of conditioned medium developed sarcomata and died within 6 weeks, the 6th dying in the 10th week.

Therefore the factor appears to have an effect on the growth of the tumour cells without any ill effect on the host. The nature of this factor and its potential use as an anti-tumour agent is being investigated.

It is a pleasure to acknowledge and thank Dr C. Milstein for his continuous interest and stimulating discussions, and for reviewing the manuscript. Also Dr J. C. Cawley for the electron microscopic examination of the cells. The work was supported by the Leukaemia Research Fund, U.K.

### REFERENCES

CAWLEY, J. C. & HAYHOE, F. G. J. (1973) *Ultrastructure of Haemic Cells*. London: W. B. Saunders Ltd.

CEROTTINI, J.-C. & BRUNNER, K. T. (1974) Cell Mediated Cytotoxicity, Allograft Rejection and Tumor Immunity. *Adv. Immun.*, **18**, 67.

GIARD, D. J., AARONSON, S. A., TODARO, G. J., ARNSTEIN, P., KERSEY, J. H., DOSIK, H. & PARKS, W. P. (1973) *In vitro* Cultivation of Human Tumours: Establishment of Cell Lines Derived from a Series of Solid Tumors. *J. natn. Cancer Inst.*, **51**, 1417.

GORDON, J., HOUGH, D., KARPAS, A. & SMITH, J. L. (1977) Immunoglobulin Expression and Synthesis by Human Haemic Cell Lines. *Immunology* (in press).

HAYHOE, F. G. J. & FLEMANS, R. J. (1969) *An Atlas of Haematological Cytology*. London: Wolfe Publishing Ltd., p. 253.

KARPAS, A. & KLEINBERG, D. (1974) Properties of Mouse Cells Transformed with DNA Containing the Murine Sarcoma Provirus. *Eur. J. Cancer*, **8**, 551.

RHIM, J. S., CHO, H. Y. & HUEBNER, R. (1975) Nonproducer Human Cells Induced by Murine Sarcoma Virus. *Int. J. Cancer*, **15**, 23.

SMITH, J. L., BARKER, C. R., CLEIN, G. P. & COLLINS, R. D. (1973) Characterisation of Malignant Mediastinal Lymphoid Neoplasm (Sternberg Sarcoma) as Thymic in Origin. *Lancet*, i, 74.